# Psychometric Properties of the Spanish Version of Psychosocial Impact of Assistive Devices Scale in a Large Sample of People With Neuromuscular, Neurological, or Hearing Disabilities

**DOI:** 10.3389/fpsyg.2021.659562

**Published:** 2021-06-10

**Authors:** Emiliano Díez, Estíbaliz Jiménez-Arberas, Thais Pousada

**Affiliations:** ^1^INICO (Institute on Community Integration), University of Salamanca, Salamanca, Spain; ^2^Occupational Therapy, Faculty Padre Ossó (Center Attached to the University of Oviedo), University of Oviedo, Oviedo, Spain; ^3^CITIC (Centre for Information and Communications Technology Research), TALIONIS (Technology Applied on Occupation, Equality and Health), University of A Coruña, A Coruña, Spain

**Keywords:** assistive technology, psychosocial impact, neurological disabilities, neuromuscular disorders, hearing disabilities, psychometric properties, outcome assesment

## Abstract

Assistive technology (AT) is any device, software, or equipment designed for and used by individuals with disabilities to engage in everyday activities and achieve independence. However, the usefulness of those technology-based or supported treatments is a complex issue that has led to the development of various conceptual models for assistive technology outcomes research and practice as well as different assessment tools that help to explore the effect of technology on people's lives. One of those instruments is the Psychosocial Impact of Assistive Device Scale (PIADS), a 26-item questionnaire that measures the psychosocial impact of interventions, using assistive devices in three quality-of-life domains: competence, adaptability, and self-esteem. PIADS scale has been translated and adapted to several languages, and it has been successfully used to measure AT outcomes in different disability profiles to predict abandonment or even as a relevant determinant of future adoption of eHealth. Quinteiro (2010) adapted PIADS to Spanish for the first time, although no studies have yet been published to systematically study its psychometric properties. Therefore, the purpose of this study was to evaluate measurement properties of the Spanish version of PIADS scale by means of a dataset obtained from its application to a large sample (*n* = 417) of people with neuromuscular, neurological, or hearing disabilities that used different assistive devices. The results will provide valuable indicators about the measurement quality of the Spanish PIADS scale and will help to promote the use of reliable and valid AT outcome assessment tools for research and clinical purposes.

## Introduction

According to ISO:9999:2016 and UNE-ISO 9999:2017, assistive technology (AT) is “any product (including devices, equipment, instruments, and software), especially manufactured or commercially available, used by or for persons with disabilities to facilitate participation; protect, support, train, measure or substitute body functions and/or structures and activities; and prevent impairments, activity limitations, or participation restrictions.” Examples of assistive devices and technologies include a variety of products as wheelchairs, prostheses, hearing aids, visual aids, communication systems, low-tech devices, and specialized software and hardware that increase mobility, hearing, vision, cognition, or communication skills, among others. Assistive devices and technologies have the primary purpose of maintaining or improving the functioning and independence of a person and, therefore, are tools that promote participation and increase the health and well-being of individual users and their families. They can also help prevent impairments and secondary health conditions. But the benefits of the use of assistive technologies are also clear in the socioeconomic field where they can serve to reduce costs in the health system or to stimulate economic growth (World Health Organization, 2018).

The Convention on the Rights of Persons with Disabilities (The United Nations, 2006) recognizes access to assistive technology as a human right and has called for international cooperation to improve access (Article 32). However, the Global Cooperation on Assistive Technology (GATE) describes that today, only 5–15% of the population that need an assistive technology have access to it, with the problem being much more serious in low- and middle-income countries (World Health Organization, 2020).

Poor availability and access to support products are complex issues determined by multiple factors. For example, Tangcharoensathien et al. (2018) consider that limited access to assistive technologies in low- and middle-income countries is determined by a variety of key factors as lack of awareness among potential beneficiaries, products designed without consideration of user preferences, economic difficulties, shortages of trained personnel, or lack of quality evidence on the effectiveness of assistive technologies. As stated by the WHO (World Health Organization, 2018), the challenges to improve access to assistive technologies are varied and involve government, professional, and industrial sectors. Thus, research and development must be improved, especially in low-income countries, and low-cost solutions must be promoted. Standards and norms must also be created to ensure the effectiveness and safety of assistive devices as well as to improve manufacturing and distribution processes.

Another major challenge is to improve services for the provision of assistive technologies. Standards must be developed to ensure quality decision-making regarding the provision of products, including assessment procedures, prescription, adjustment, user training, follow-up, maintenance, and repair. In this context, it is of great importance to have adequate models, and the corresponding assessment tools that help to make decisions and evaluate outcomes in interventions with assistive devices. A review of such models is beyond the scope of this paper [see, for example, Lenker and Paquet (2003); Bernd et al. (2009); Alves et al. (2016); Federici and Scherer (2018)]. But the literature in recent years has showed some assessment instruments that have proved to be particularly useful, as it is the case of the Psychosocial Impact of Assistive Devices Scale (PIADS; Jutai and Day, 2002; Day and Jutai, 2003).

The PIADS scale was designed to measure the user's perception of the impact of assistive technology on his/her functional independence, well-being, and quality of life with 26 self-rated items grouped into three perspectives. Twelve items measure the feelings of competence and efficacy associated with the use of a product (competence); six items are related to the willingness to try out new things and to take risks (adaptability); and eight items measure feelings of emotional health and happiness (self-esteem). The PIADS scale has been translated to 19 languages, and it has been used in more than 120 scientific works to evaluate results derived from interventions with a huge variety of products and assistive technologies as well as a wide range of user profiles (Jutai, 2019). It also has proved to have good reliability and content validity, excellent internal consistency, and structural, cross-cultural, and criterion validity (de Lima Barroso et al., 2018), and seems to be powerful enough to predict assistive technology abandonment and retention (Day et al., 2001, 2002).

Two Spanish PIADS versions are available: one for Puerto Rican-Spanish (Orellano and Jutai, 2013; Orellano-Colón et al., 2016) and the other for Spain-Spanish (Quinteiro, 2010). To our knowledge, no studies have yet been published to systematically explore the psychometric properties of the Spain-Spanish PIADS version. Bearing in mind that construct validity and reliability are critical aspects in order to ensure a good adaptation of an assessment instrument, this work aims to study the psychometric properties of the Spanish-PIADS from its application to a large sample of people with neuromuscular, neurological, or hearing disabilities, using a variety of assistive devices. More specifically, the objectives of the research were to analyze the internal consistency and the factorial structure (confirmatory factor analysis) of the Spanish-PIADS.

## Methods

### Sample

The sample was composed of 417 adults who had been administered the Spanish version of the PIADS scale in other independent studies (Pousada et al., 2015; Jiménez-Arberas et al., 2019; Jiménez-Arberas and Díez, 2021). In all cases, a convenience sampling was used, and the scale had been applied as a way of assessing the perceived psychosocial impact related to the use of the person's main assistive technology. Table 1 shows the main sample characteristics as a function of the type of disability.

**Table 1:** Socio-demographic characteristics of the sample by the type of disability.

	**All sample**	**Acquired**	**Hearing loss**	**Neurodegenerative**	**Neuromuscular**	**Other diagnoses**
		**brain injury**		**diseases**	**disorders**	
**Sex (*****N*****)**						
Female	231	23	175	6	22	5
Male	186	27	113	14	38	4
**Age (years)**						
Mean (*SD*)	55.2 (23)	59 (15.2)	56.6 (24.9)	55.5 (13.6)	43.8 (16.1)	72.4 (20.4)
**Assistivetechnology (*****N*****)**						
Behind The Ear (BTE) hearing aid	90		90			
Cochlear implant	30		30			
Completely In the Canal (CIC) hearing aid	36		36			
Deep insertion hearing aid	5		5			
Hearing glasses	2		2			
Instant voice and text messaging app (Oovoo)	17		17			
Software Skype	15		15			
Video Relay Service (Svisual)	66		66			
External Ear Sound Amplifier	1		1			
Powered Wheelchair	54	14		8	32	
Manual Wheelchair	41	7		7	25	2
Mobile Phone	26		26			
Quad Cane Walking Stick	1					1
Trekking cane	8	8				
Walker	8	2		2	1	3
Crutch	16	9		2	2	3
Foot-Up	1			1		

### Measures

The Spanish version of the PIADS scale adapted by Quinteiro (2010) was used in this study. The adaptation followed the instructions provided by the authors of the original instrument and roughly consisted of translating and adapting to Spanish the original questionnaire, the glossary of terms, the spreadsheet of the results, and the guidelines for application. Subsequently, a backward translation into English was carried out, which was reviewed and approved by the authors of the original scale.

### Procedure

The application of the PIADS scale was carried out through different methods, including interviews by experienced occupational therapists (85.9%), questionnaires sent by post/e-mail (5%), and a self-administered webform version of the scale (9.1%).

The administration procedure of the PIADS scale consisted of showing a list of words or short phrases describing how the use of an assistive device may affect a person (e.g., willingness to take chances, independence, or self-confidence). For each word/short sentence, the participants rated the extent to which they were affected, using a seven-point Likert scale, ranging from −3 (maximum negative impact) to +3 (maximum positive impact) with a 0 midpoint, indicating no impact or no perceived change as a result of using the assistive device. Following scale completion instructions, if the participant asked for a definition for a PIADS item, the experimenter gave the explanation for the item taken from the PIADS glossary.

### Analysis

Analyses were performed with JAMOVI (The jamovi project, 2020) and R software (v.4.0.2) (R Core Team, 2020) by using, mainly, lavaan (v. 0.6-7) (Roseel, 2012), semTools (v.5.4) (Jorgensen et al., 2021), psych (v. 2.0.12) (Revelle, 2020), boot (v. 1.3-25) (Davison and Hinkley, 1997; Canty and Ripley, 2020), and EFA tools (v.3.0) (Steiner and Grieder, 2020) packages.

Confirmatory factor analysis (CFA) was performed, using the MLMV estimator, and the following several indices and cutoffs criteria were used to analyze the goodness of the data fit by the different models: comparative fit index (CFI ≥ 0.90) as incremental fit indices, and standardized root mean square residual (SRMR <0.08) and root mean squared error of approximation (RMSEA <0.05) as baseline fit indices. Two information criteria (the AIC and the BIC) were also computed.

## Results

### Psychosocial Impact by Subscale and Disability Groups

Descriptive results showed (Table 2) that the psychosocial impact of assistive devices perceived by the participants was mainly positive, with positive mean scores for the three subscales. A mixed analysis of variance (ANOVA) with one within-subjects factor with three levels (psychosocial impact, PIADS: competence, adaptability, and self-esteem) and one between-subjects factor (disability group: hearing, neuromuscular, or neurological disability) showed a significant main effect of psychosocial impact [*F*_(1.80, 745.5)_ = 68.70; *p* < 0.001; η^2^-*p* = 0.14] as well as a significant interaction psychosocial impact x disability [*F*_(3.60, 745.5)_ = 9.49; *p* < 0.001; η^2^-*p* = 0.04]. *Post-hoc* (Bonferroni corrected) comparisons showed significant greater scores for competence than for adaptability [*t*_(828)_ = −5.97; *p* < 0.001; *d* = – 0.31, 95% CI (– 0.41, – 0.21)] and self-esteem [*t*_(828)_ = 5.75; *p* < 0.001; *d* = 0.24, 95% CI (0.14, 0.34)], and also greater adaptability than self-esteem [*t*_(828)_ = 11.72; *p* < 0.001; *d* = 0.43, 95% CI (0.32, 0.53)] for the whole sample. For competence or adaptability, no differences were observed as a function of disability group, but, in the case of self-esteem, significant greater scores were found for the group of hearing disabilities in comparison to neuromuscular [*t*_(546)_ = 3.25; *p* < 0.001; *d* = 0.45, 95% CI (0.18, 0.72)] or neurological groups [*t*_(546)_ = 4.93; *p* < 0.001; *d* = 0.65, 95% CI (0.37, 0.92)].

**Table 2:** Mean, standard deviations, minimum, maximum scores in each subscale of the Spanish (Spain) Psychosocial Impact of Assistive Device Scale by the disability group.

**PIADS subscale**	**Mean**	***SD***	**Min**	**Max**
**All sample (*****n*** **=** **417)**				
Competence	1.13	1.03	−2.08	3.00
Adaptability	1.32	1.11	−2.17	3.00
Self-steem	0.98	1.11	−2.75	3.00
**Hearing disabilities (*****n*** **=** **288)**				
Competence	1.24	0.99	−1.66	3.00
Adaptability	1.38	1.08	−2.17	3.00
Self-steem	1.16	1.21	−2.00	3.00
**Neuromuscular disorders (+** **5 from other diagnoses: 2 Spinal**				
**cord injury, 2 Spina Bifida, 1 Sudeck Syndrome) (*****n*** **=** **65)**				
Competence	1.00	0.84	−1.00	2.92
Adaptability	1.21	1.04	−0.83	3.00
Self-steem	0.68	0.78	−1.00	2.25
**Neurological disabilities (acquired brain injury, neurodegenerative**				
**diseases**, **+** **4 from other diagnoses -cerebral palsy-) (*****n*** **=** **64)**				
Competence	0.77	1.28	−2.08	2.75
Adaptability	1.18	1.29	−2.00	3.00
Self-steem	0.43	1.13	−2.75	2.25

### Validity Evidence Based on Internal Structure

To our knowledge, no published studies have assessed the factorial structure of PIADS against the proposed three-factor model that has been extensively used to interpret PIADS scale applications. In this study, we used confirmatory factor analysis (CFA) models to compare the proposed original factorial structure solution (three correlated factors: competence, adaptability, and self-esteem), with different competing models that could also explain the PIADS factorial structure: a single-factor model for testing the key assumption of unidimensionality; a three-uncorrelated-factor model with the same structure as the original (competence, adaptability, and self-esteem); a higher-order model, which incorporates a superordinate global psychosocial impact factor mediated by a series of subordinates factors (competence, adaptability, and self-esteem); and a bifactor model, including a general factor that loads directly onto all items and three grouping factors (competence, adaptability, and self-esteem), which load onto specific items for those subscales for testing orthogonality of the factors with a general factor.

As reported in Table 3, model χ^2^, which assesses the overall fit and the discrepancy between the sample and fitted covariance matrices, resulted in rejecting the null hypothesis of the perfect model fit for all models. However, due to its sensitivity to sample size, χ^2^/df ratio was also considered. The ratios were <3 for all models, except for model C.

**Table 3:** Confirmatory factor analysis (CFA) model fit summary.

	**χ2 (df)**	**χ2/df**	**RMSEA**	**CFI**	**SRMR**	**AIC**	**BIC**
Model A	585.991 (296)^***^	1.98	0.048	0.882	0.057	33,405.955	33,732.635
Model B	605.116 (299)^***^	2.02	0.050	0.876	0.058	33,451.851	33,766.431
Model C	1033.716 (299)^***^	3.46	0.077	0.701	0.361	34,602.415	34,916.995
Model D	592.026 (298)^***^	1.99	0.048	0.880	0.061	33,411.335	33,729.948
Model E	689.111 (297)^***^	2.32	0.056	0.841	0.185	33,707.867	34,030.513
Model F	438.302 (227)^***^	1.93	0.047	0.909	0.045	28,280.971	28,571.354
Model G	456.093 (230)^***^	1.99	0.049	0.903	0.046	28,324.100	28,602.383

*** *p < 0.001*.

*(A) The original model, three correlated factors (competence, adaptability, and self-esteem); (B) the single-factor model; (C) the three-uncorrelated-factor model (competence, adaptability, and self-esteem); (D) the higher-order model; (E) the bifactor model; (F) three correlated factors with no reversed items; (G) the single-factor model with no reversed items*.

*AIC, Akike information criteria; BIC, Bayesian information criteria; CFI, comparative fit index; RMSEA, root mean square error of approximation; SRMR, standardized root mean square residual*.

In relation to the models that included all the items of the original scale (models A–E), the measures of the model fit showed the best results for the three-correlated-factor model (model A) and the single-factor model (model B), with measures denoting almost acceptable (CFI very close to 0.90), acceptable (SRMR <0.08) and a good fit (RMSEA ≤ 0.05).

**Figure 1:**
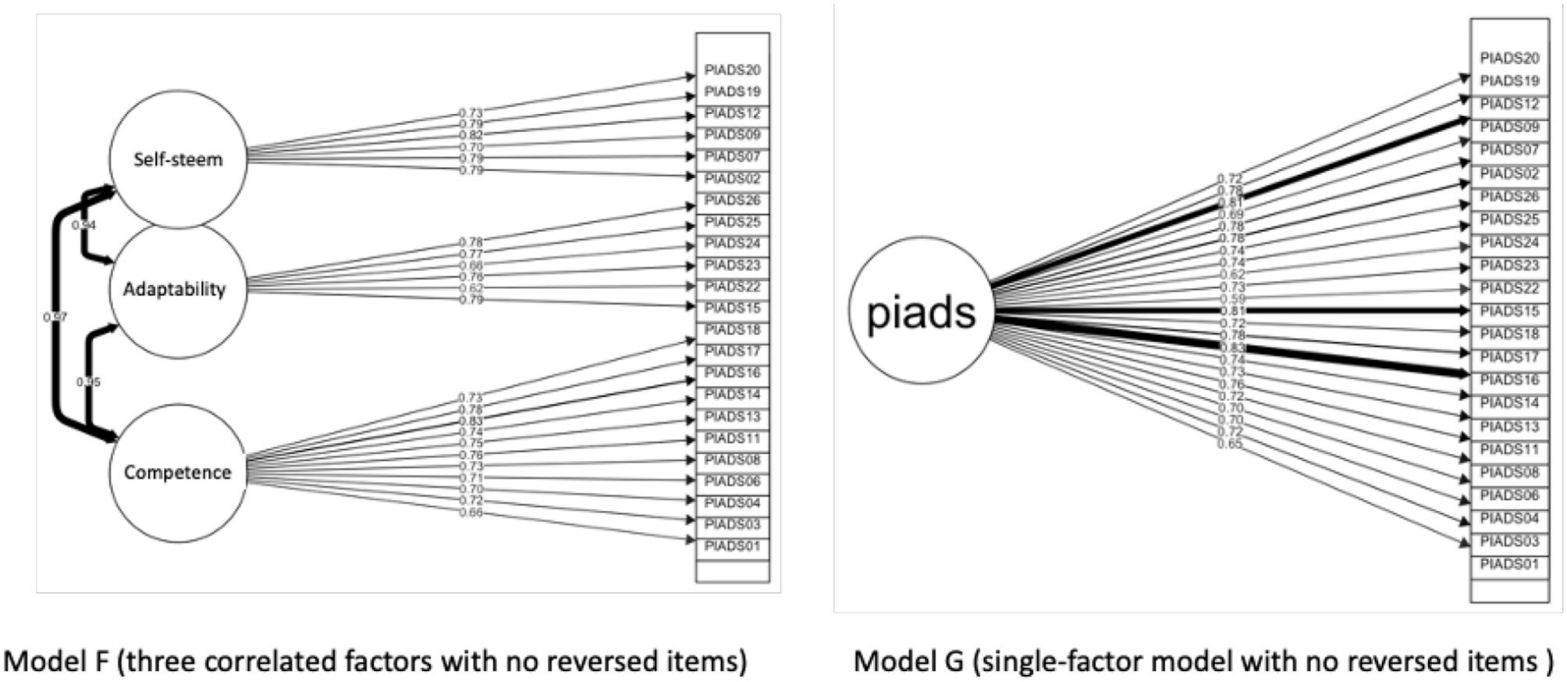
Confirmatory Factor Analysis (CFA) of PIADS Scale with no reversed items. Fit indexes (see Table 3) showed the best results for this two models.

The standardized factor loadings for the most commonly PIADS factor model (three correlated factors) showed values ranging from 0.62 to 0.83 for 23 items. But, for three items, the loadings were especially low (items 5, 10, and 21, with loadings of 0.11, 0.23, and 0.16, respectively). We also explored the local misfit with a residual variance–covariance matrix, and those three items exhibited very high positive residual covariances. The affected items belonged to the competence (item 5, confusion) and self-esteem (item 10, frustration; and 21, embarrassment) factors. Precisely, those items are the only ones in the PIADS scale that are reversed (higher positive scores denoting worse impact). We decided to create two new models (single-factor and three correlated factors), deleting those items (Models F and G in Table 3; see also Figure 1). This time, all the measures showed a good fit to both the single-factor and the three-correlated-factor models (CFI ≥ 0.90; SRMR < 0.05; RMSEA < = 0.05), denoting a potential problem with the differential response format of these three items. The average variance extracted (AVE) for the F model showed values >0.5 for all PIADS subscales (competence = 0.55; adaptability = 0.54; self-esteem = 0.60), denoting acceptable convergent validity.

### Internal Consistency

When considering the original complete scale, model comparisons revealed the superiority of the original three-correlated-factor solution. Consequently, this factorial solution was chosen to perform reliability analysis. The reliability of each of the three factors was determined, using ordinal McDonald's omega and Cronbach's alpha (Elosua and Zumbo, 2008; Peters, 2014). Also, nonparametric confidence intervals (CI) were estimated with a bootstrap procedure, using the adjusted bootstrap percentile (BCa) method. As shown in Table 4, both Cronbach's Alpha and McDonald's Omega showed excellent internal consistency for the whole scale and the competence subscale, and good for adaptability and self-esteem subscales. When those indexes were also calculated for the model with no reversed items, an increment in both Cronbach's alpha and McDonald's omega was verified for the competence (α = 0.93; ω = 0.93) and self-esteem (α = 0.90; ω = 0.90) factors.

**Table 4:** Ordinal Cronbach's alpha and McDonald's omega (with bootstrap confidence intervals) scores in each subscale of the Spanish (Spain) Psychosocial Impact of Assistive Device Scale.

**PIADS subscale**	**Crobach's α (95% CI)**	**McDonald's ω (95% CI)**
Competence	0.93 (0.92–0.94)	0.94 (0.93–0.94)
Adaptability	0.90 (0.89–0.92)	0.90 (0.89–0.92)
Self-steem	0.87 (0.85–0.89)	0.88 (0.87–0.90)
Global Score	0.96(0.96–0.97)	0.97(0.96–0.97)

## Discussion

The present research aimed to obtain psychometric evidence for the use of the PIADS scale in Spain. The need for standardized methods for the follow-up of individual interventions with AT, especially through outcome measures that show good metric properties, motivated the exploration of the factorial structure and internal consistency of the Spanish PIADS scale based on data from its application to a large sample of participants, using different assistive devices.

First, because the study of dimensionality considerations is very important when reporting scores in order to assess more specifically the psychosocial impact of using assistive technologies, we were interested in analyzing in detail the structural validity of the Spanish PIADS. Previously, other studies reported evidence for construct validity by way of exploratory factorial analysis, mainly using Principal Component Analysis. For example, Jutai and Day (2002) found a solution of three factors, accounting for 61.1% of the total variance. But this is the first time that Confirmatory Factor Analysis has been used to test both the validity and the reliability of the PIADS scale. Specifically, we have compared the factorial structure of the original PIADS scale (three correlated factors) with other possible alternative structures. The results have shown acceptable fit measures for both a single factor and three correlated factors, although slightly favoring the latter but showed worst results for other common factorial structures as the higher-order model or the bifactor model.

Second, reliability analysis based on the three-factor structure showed that PIADS has a very good internal consistency, confirming the results of many other studies adapting PIADS to other languages (e.g., Chae and Jo, 2014; Tofani et al., 2020), although showing worst results for the self-esteem subscale as has also been verified in other adaptation studies (e.g., Demers et al., 2002; Hsieh and Lenker, 2006).

Third, the detailed exploration of the item loadings showed some problems with the items that are reversed in the PIADS scale. When fitting both single-factor and three-correlated-factor models with those items deleted, all model fit measures, as well as internal consistency measures, increased. Reversed items are common means of controlling for the effects of acquiescence, but, usually, in the context of balanced scales, where half of the items measure the construct in one direction and, the other half, in the opposite direction. In the PIADS scale, only three items (out of 26) are reversed, and this could favor the negative effects of having reversed items. There is evidence that the use of reversed items may have positive effects (e.g., increase validity by providing a more complete representation of an underlying construct or promoting more careful reading of the items). But it also could have negative effects, as the reduction of internal consistency and affectation to the factorial structure of measures, being common to observe a poor fit to the expected model (Vigil-Colet et al., 2020). Although there is no agreement in the literature regarding the use of reversed items, in the case of the PIADS scale, our results could suggest to change the direction of the reversed items or, alternatively, to include more reversed items to maintain the equivalent proportion of positive and negative items in each subscale.

Fourth, the analysis of differences in the PIADS scores has shown a general positive psychosocial impact associated with the use of different assistive technologies in a heterogeneous sample of participants with disabilities. Specifically, the impact has been greater for aspects related to perceived functional capability, independence, and performance (i.e., competence) in comparison to other aspects like the inclination or motivation to participate socially and take risks (i.e., adaptability) or the perceived self-confidence, self-esteem, and emotional well-being (i.e., self-esteem). The scores on the self-esteem dimension were the lowest, in line with the results of other recent studies (e.g., Devitt et al., 2004; Orellano-Colón et al., 2016; Pousada et al., 2021). Likewise, significant differences were verified in the dimension of self-esteem as a function of a disability group, finding better values for the participants with hearing impairment compared with the participants with neurological or neuromuscular disabilities.

In addition to all these results, other studies with the Spanish PIADS version (Jiménez-Arberas and Díez, 2021) have demonstrated the predictive validity of the Spanish version of the PIADS scale on the abandonment and retention of assistive technologies (e.g., a correlation of −0.54 to −0.61 with abandonment), as well as a moderate but reliable correlation with the history of positive experiences with technology as measured by the Matching Person and Technology instrument (Scherer, 2002).

This study has some limitations, in particular the use of samples of the participants chosen by convenience sampling procedures and, also, the small sample size of some disability groups and the limited disability profiles considered. With random sampling and a higher and more balanced sample size across disability groups, age, and sex, it would be possible to study the PIADS subscale's measurement invariance, which plays a crucial role in the interpretation of test scores appropriately for individuals from different populations or cultures. It would also have been of interest to administer other measures to the entire sample (e.g., quality of life) in order to analyze the convergent validity of the PIADS, as well as following-up participants to determine the predictive validity of each factor on the possible future abandonment of the assistive devices. These limitations point to future research lines.

In summary, our results build on existing evidence of the good to excellent psychometric properties of the PIADS scale and corroborate the possibility of using it in subsequent studies as a valid and reliable outcomes measure of the psychosocial impact of assistive technology users. In addition, other recent results have also shown the compatibility of the PIADS language with models of human functioning frequently used in the rehabilitation field, such as the International Classification of Functioning, Disability and Health (ICF), which strengthens the potential implementation of PIADS in those contexts (Traversoni et al., 2018). Also, the three PIADS dimensions have proved their usefulness as relevant determinants of the adoption of eHealth solutions in the elderly (Axelsson and Wikman, 2016). All this evidence, undoubtedly, can contribute significantly to better inform the usefulness of the technology-based or supported interventions and thus to improve the quality of life of Spanish-speaking people with disabilities.

## Data Availability Statement

The data analyzed in this study is subject to the following licenses/restrictions: The raw data supporting the conclusions of this article will be made available by the authors, on reasonable requests. Requests to access these datasets should be directed to Emiliano Díez, emid@usal.es.

## Ethics Statement

All procedures performed in studies involving human participants were in accordance with the ethical standards of the institutional and/or national research committee and with the 1964 Helsinki Declaration and its later amendments or comparable ethical standards. Ethical review and approval was not required for the study on human participants in accordance with the local legislation and institutional requirements. The patients/participants provided their written informed consent to participate in this study.

## Author Contributions

ED: conceptualization, methodology, formal analysis, data curation, writing the original draft, writing, reviewing, editing, and project administration. EJ-A: conceptualization, investigation, resources, data curation, writing, reviewing, and editing. TP: conceptualization, investigation, resources, data curation, writing, reviewing, and editing. All authors contributed to the article and approved the submitted version.

## Conflict of Interest

The authors declare that the research was conducted in the absence of any commercial or financial relationships that could be construed as a potential conflict of interest.
